# Prognosis of premenopausal women with low-risk endometrial cancer but elevated CA125 levels

**DOI:** 10.3389/fonc.2024.1510988

**Published:** 2025-01-21

**Authors:** Jeong Min Song, Ala Aiob, Kidong Kim, Kwang Beom Lee, Sokbom Kang, Chae Hyeong Lee, Se Ik Kim, Nam Kyeong Kim, Dae Hoon Jeong

**Affiliations:** ^1^ Department of Obstetrics and Gynecology, Kyung Hee University School of Medicine, Kyung Hee University Hospital at Gangdong, Seoul, Republic of Korea; ^2^ Department of Obstetrics and Gynecology, Galilee Medical Center, Nahariya, Israel; ^3^ Azrieli Faculty of Medicine, Bar Ilan University, Safed, Israel; ^4^ Department of Obstetrics and Gynecology, Seoul National University Bundang Hospital, Seongnam, Republic of Korea; ^5^ Department of Obstetrics and Gynecology, Seoul National University College of Medicine, Seoul, Republic of Korea; ^6^ Department of Obstetrics and Gynecology, Gachon University Gil Medical Center, Gachon University College of Medicine, Incheon, Republic of Korea; ^7^ Center for Gynecologic Cancer, National Cancer Center, Goyang, Republic of Korea; ^8^ Department of Obstetrics and Gynecology, Graduate School of Medicine, Dongguk University, Seoul, Republic of Korea; ^9^ Department of Obstetrics and Gynecology, Busan Paik Hospital, Paik Institute for Clinical Research, Inje University, Busan, Republic of Korea

**Keywords:** endometrial cancer, lymphadenectomy, CA125, premenopausal, lymphatic metastasis

## Abstract

**Introduction:**

Women with low-risk endometrial cancer, as defined by the Korean Gynecologic Oncology Group (KGOG) criteria, have a low risk of lymph node metastasis and an excellent prognosis without lymphadenectomy. However, it is unclear whether lymphadenectomy should be performed in premenopausal women who meet the KGOG criteria other than elevated cancer antigen 125 (CA125) levels, because the CA125 level can be elevated by benign conditions. We investigated the patterns of metastasis and recurrence to assess the value of lymphadenectomy in this population.

**Methods:**

Premenopausal women with endometrial cancer meeting the KGOG criteria, except for those with elevated CA125 levels, were eligible. The characteristics of the eligible women were collected from seven institutes in the Republic of Korea by reviewing their medical records. Recurrence-free survival (RFS) was estimated using the Kaplan–Meier method and compared using the log-rank test.

**Results:**

Seventy-three patients were included. Of 62 women who underwent lymphadenectomy, only two (3.2%) had lymph node metastasis. Eighteen women (24.7%) received adjuvant therapy. At a median follow-up of 59 months, the 5-year RFS was 88.8%. Five women (7%) experienced recurrence, two had lymph node recurrence, and three had non-nodal recurrence. RFS was similar between the women who did and did not undergo lymphadenectomy (P=0.737).

**Conclusion:**

Premenopausal women who had elevated CA125 levels but met all other KGOG criteria showed a low risk of lymph node metastasis and recurrence as well as a good prognosis. Therefore, lymphadenectomy can be omitted in this population.

## Introduction

Endometrial cancer is the most common female genital tract malignancy in Western countries (1). Approximately 73% of patients with endometrial cancer are diagnosed at stage I, resulting in a good prognosis, with a 5-year overall survival rate of 85–91% (2).

The conventional initial approach for women with newly diagnosed endometrial cancer involves surgical staging with total hysterectomy, bilateral salpingo-oophorectomy, lymph node (LN) examination, and extrauterine disease evaluation. Owing to the low rate of lymph node metastasis (LNM) in patients with early-stage endometrial cancer (3), it is debatable whether lymphadenectomy is necessary in all patients with early endometrial cancer (3–5).

Lymphadenectomy leads to higher intra- and postoperative complications, prolonged operative time, and hospitalization days (6, 7), and does not improve survival compared with no lymphadenectomy for low-risk endometrial cancer stage I (8). In two randomized clinical trials of low-risk endometrial cancer, lymphadenectomy did not affect survival and caused more complications (6, 7). However, despite insufficient evidence supporting routine lymphadenectomy in low-risk patients, many surgeons still perform lymphadenectomy for all endometrial cancers, regardless of the tumor stage or characteristics (9–11).

Several models have been proposed to predict the likelihood of LNM using histopathological parameters in women with endometrial cancer. In a Mayo Clinic series, patients with low-risk endometrial cancer had 5% LNM with no LN involvement in tumors <2 cm (12). Other retrospective analyses demonstrated similar results in patients with low-risk characteristics (13–17). Another multicenter study of women with low-risk endometrial cancer conducted by the Korean Gynecologic Oncology Group (KGOG) reported that the likelihood of LNM in this group was 2% or lower (18). Based on these findings, it is reasonable to omit lymphadenectomy in the low-risk subgroup.

A high cancer antigen 125 (CA125) level (>35 IU/mL) is associated with a poor prognosis (19). The risk of LNM increased from 15.9% to 45.7% when the CA125 level was >35 IU/mL. Preoperative CA125 levels are significantly associated with the extent of disease and LNM (20–23). However, elevated CA125 levels in premenopausal women are reported to have approximately a 5% of false-positive results, which can be attribute to benign conditions such as uterine fibroids (24), endometriosis, or adenomyosis, rather than metastasis of endometrial cancer to the LNs (24–26). Therefore, there is a concern about performing lymphadenectomy in low-risk perimenopausal women with elevated CA125 levels. Moreover, there are insufficient data about LN involvement in endometrioid cancer in premenopausal women with high CA125 levels, particularly in the absence of other risk factors for LN involvement, such as non-endometrioid histology, deep myometrial invasion, positive peritoneal cytology and/or lymphovascular space invasion (27). Therefore, this study aimed to verify whether lymphadenectomy should be performed for ultra-low-risk endometrial cancer, as defined by the KGOG (18) in premenopausal patients with elevated CA125 levels.

## Materials and methods

### Ethics statement

This study was initially approved by the Seoul National University Bundang Hospital Institutional Review Board (SHUB-2212-799-101) Institutional Review Boards of each participating institution. The requirement for informed consent was waived owing to the retrospective nature of this study.

### Study design and population

From the institutions’ endometrial cancer cohorts, we retrospectively identified patients who met the following conditions: (1) diagnosis of primary endometrioid endometrial cancer between January 2013 and December 2021; (2) no evidence of LN, pulmonary, or other distant metastases on computed tomography (CT), magnetic resonance imaging (MRI), positron emission tomography (PET), or PET/CT within 2 months before hysterectomy (LNs measuring > 1 cm in the short-axis diameter on CT and MRI were considered metastases. On PET/CT, the preoperative diagnosis of LNM was based on the report from the nuclear medicine radiologist); (3) preoperative pelvic MRI scans within 2 months before hysterectomy showing no evidence of myometrial invasion of >50%, cervical or extrauterine involvement, and LNM; (4) receipt of comprehensive surgical staging included total hysterectomy, bilateral salpingo-oophorectomy, and cytology; (5) premenopausal status with serum CA125 levels >35 IU/mL within 2 months before hysterectomy. Patients who underwent chemotherapy, radiotherapy, or progestin treatment for endometrial cancer before the primary surgery and those with a history of cancer history within 5 years of endometrial carcinoma diagnosis were excluded from the analysis.

### Data collection

All MRI scans were performed using 1.5-T or 3.0-T magnets after gadolinium enhancement. The acquired imaging parameters included deep myometrial invasion, LN size, and extension beyond the uterine corpus. A cutoff value of 35 IU/mL was used to identify abnormally elevated blood CA125 levels (21, 22).

The choice of adjuvant therapy and surveillance methods followed the treatment protocols of each medical center, guided by organizations such as the Korean Society of Gynecologic Oncology and the National Comprehensive Cancer Network, with due consideration of individual patient factors and preferences.

From the medical records and pathologic reports, we retrieved baseline demographics and clinical characteristics, including the 2009 International Federation of Gynecology and Obstetrics (FIGO) stage, histologic grade, serum CA125 levels, number of removed and involved LNs, status of LNs, and type of adjuvant therapy after surgery.

### Statistical analyses

The Student t-test and Mann–Whitney U test were used to compare continuous variables. Pearson chi-square and Fisher exact tests were used to compare categorical variables. Survival outcomes were compared between the two groups using the Kaplan–Meier method with the log-rank test. All statistical analyses were performed using SPSS Statistics software (version 25.0; IBM Corp., Armonk, NY, USA). Statistical significance was set at P<0.05.

## Results

The study cohort comprised 73 premenopausal women with low-risk endometrial carcinoma and elevated CA125 levels. The median age was 48 (range, 31–59) years, median body mass index was 24.3 (range, 17.7–44.9) kg/m^2^, and mean CA125 level was 51.0 (standard deviation, 66.1) IU/mL. Most women had endometrioid type (97.3%) on the final histology. Subsequent hysterectomy grading revealed that grade 1 tumors were the most prevalent (57.5%), followed by grades 2 (27.4%) and grade 3 (6.8%). The distribution of patients by FIGO stage was as follows: IA, 75.3%; IB, 12.3%; II, 5.5%; III, 5.5%; and IV, 1.4%.

Lymphadenectomy was conducted in most patients (84.9%). The mean number of LNs removed was approximately 19.8 ± 19.3. Among women who underwent lymphadenectomy (62), two women (3.2%) exhibited LN metastasis on pathology (**Table 1**). It is important to note that patients who did not undergo lymphadenectomy had no enlarged LNs detected on imaging. The administration of adjuvant treatment varied within the cohort; 75.3% (55) of patients did not receive any adjuvant treatment. Seven women (9.6%) received radiation therapy, 2 (2.7%) received concurrent chemoradiation therapy, and 9 (12.3%) received chemotherapy.

**Table 1:** Baseline characteristics.

Baseline characteristics (n=73)	Value
Age (mean ± SD)	46.5 ± 5.9
BMI (mean ± SD)	25.9 ± 6.0
Preoperative CA125 level, IU/mL (mean ± SD)	77.4 ± 66.1
Preoperative grade on endometrial biopsy, n (%)	
1	49 (67.1)
2	11 (15.1)
3	6 (8.2)
Unknown	7 (9.6)
Lymphadenectomy, n (%)	
No	11 (15.1)
Yes	62 (84.9)
Number of removed nodes (mean ± SD)	19.8 ± 19.3
Histology type at hysterectomy specimen, n (%)	
Endometrioid	71 (97.3)
Others*	2 (2.7)
Grade on hysterectomy, n (%)	
1	42 (57.5)
2	20 (27.4)
3	5 (6.8)
Unknown	6 (8.2)
FIGO stage, n (%)	
IA	55 (75.3)
IB	9 (12.3)
II	4 (5.5)
III	4 (5.5)
IV	1 (1.4)
LN metastasis on pathology**, n (%)	
No	60/62 (96.8)
Yes	2/62 (3.2)
Adjuvant treatment, n (%)	
No	55 (75.3)
Radiotherapy	7 (9.6)
CCRT	2 (2.7)
Chemotherapy	9 (12.3)
Total recurrence, n (%)	5 (6.8)
LN recurrence, n (%)	2 (2.7)
Another site, n (%)	3 (4.1)
Death n (%)	3 (4.1)

* Non atypical hyperplasia; ** excluding 11 patients without lymph node removal

CCRT, concurrent chemoradiation therapy; CA125, cancer antigen 125; SD, standard deviation; BMI, body mass index; LN, lymph node; FIGO, 2009 International Federation of Gynecology and Obstetrics

**Figure 1:**
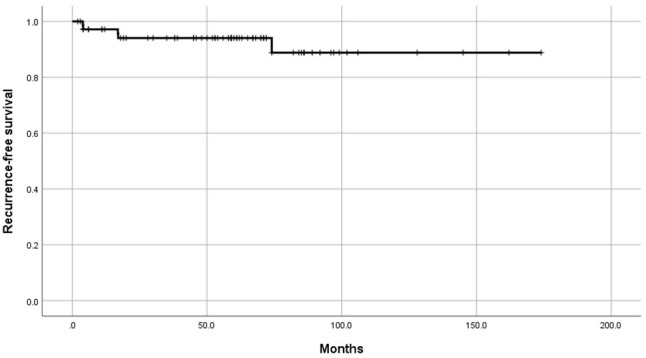
Kaplan–Meier recurrence-free survival curves for the whole cohort.

Regarding patient and recurrence-free survival (RFS), the median follow-up duration was 59 (2–174) months. Five (6.8%) recurrences and three (4.1%) deaths were recorded. The rate of 5-year RFS was 88.8% (**Figure 1**).

Among five patients who experienced recurrence, two (2.7%) involved LNs, whereas the remaining three showed recurrence at other sites. None of the five women had LNM at the time of primary treatment.

**Figure 2:**
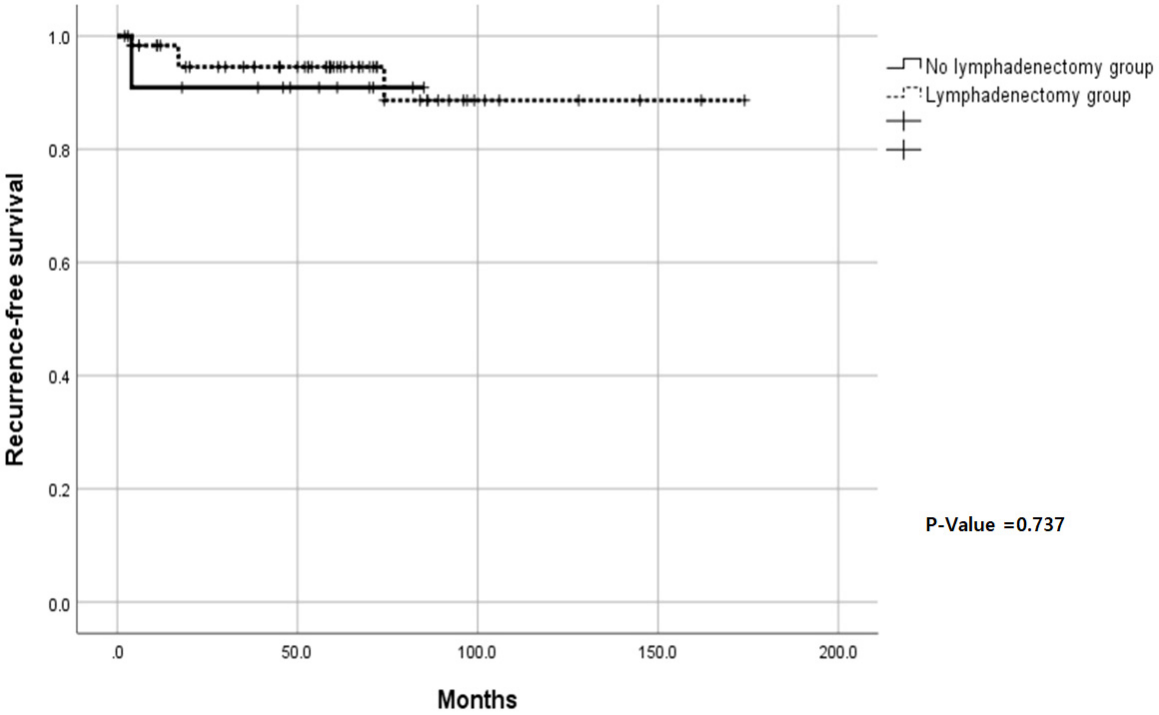
Kaplan–Meier recurrence-free survival curves for the lymphadenectomy and non-lymphadenectomy groups (Log-rank P-value: 0.737).

There were no significant differences in the recurrence rate and RFS between women who did and did not undergo lymphadenectomy (6.5% and 9.1%, respectively; P=0.569), and RFS analysis showed no significant difference in the 5-year PFS (P=0.737) (**Figure 2**).

Five women in our cohort (6.8%) had endometrioid-type carcinoma grade 3 on final histology. All five of them underwent hysterectomy, bilateral salpingo-oophorectomy, and lymphadenectomy. Their staging revealed two cases of stage IA, two cases of stage II, and one case of stage IIIB. Notably, no LNM was observed in any case. A single recurrence was observed. Recurrence occurred in a 36-year-old woman with an initial CA125 level of 46 IU/mL at the time of diagnosis. She was diagnosed with stage II and she did not undergo adjuvant treatment. Recurrence occurred in the surgical stump a year after the initial treatment.

## Discussion

This study investigated the need for lymphadenectomy in patients with low-risk endometrial cancer, as defined by the KGOG, who also have elevated CA125 levels. Our study highlighted several significant findings that contribute to the ongoing debate on the necessity of lymphadenectomy in this patient population.

First, the rate of LN metastasis in premenopausal women with low-risk endometrial cancer and elevated CA125 levels was 3.2%. This is consistent with the results of previous studies. In a series from the Mayo Clinic, 187 patients with low-risk endometrial cancer underwent comprehensive lymphadenectomy as part of their surgical staging. Only nine of 187 (5%) had evidence of LNM. Within this low-risk group, there were no patients with LN involvement if the tumor size was <2 cm (12). Another cohort of women with low-risk endometrial cancer was examined in a multicenter study conducted by KGOG. This cohort comprised patients who did not meet the following criteria on MRI: deep myometrial invasion, enlarged LNs, extension beyond the uterine corpus, or CA125 levels exceeding 35 IU/mL. The likelihood of LNM in this specific group was≤2% (18). Notably, our study and the KGOG study (18) share similar criteria except for elevated CA125 levels.

**Figure 3:**
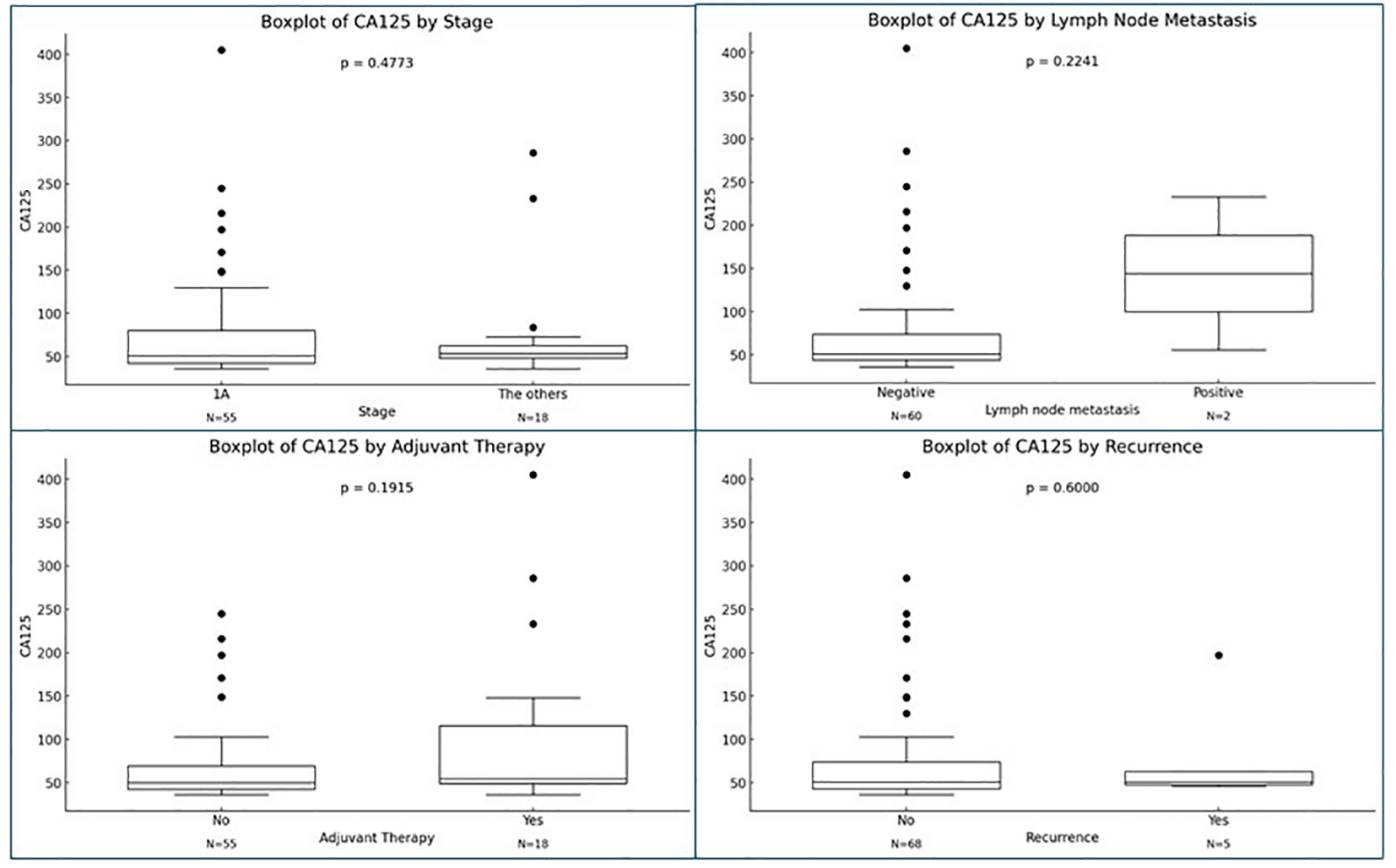
Association of CA125 level with various variables. Lymph node was not removed in 11 patients. P-value was calculated using Mann-Whitney U test.

This suggests that in our specific patient population, the likelihood of LN involvement was minimal, and the elevated CA125 level in this low-risk group of women had no effect on the rate of LNM. We acknowledge that elevated CA125 levels in premenopausal patients can be caused by endometriosis or adenomyosis (25), which may not necessarily indicate metastasis of endometrial cancer to the LNs. As, illustrated in **Figure 3**, although elevated CA125 levels appear to correlate with prognostic factors indicative of worse outcomes, such as advanced stage and lymph node metastasis, these associations did not achieve statistical significance, likely due to the limited sample size (N). However, it is essential to highlight that in a single study encompassing both low- and high-risk groups of women, an elevated CA125 level (>35 IU/mL) was associated with a poorer prognosis and an increased risk of LNM (15.9% to 45.7%) (20).

Second, the median follow-up duration was 59 months, and the RFS rate observed in our study was 89%, which underscores the generally favorable prognosis for premenopausal patients with low-risk endometrial cancer, even with elevated CA125 levels. Additionally, no significant differences were found in the recurrence rate and the 5-year RFS among women who did and did not undergo lymphadenectomy. This RFS rate is consistent with those in previously reported research in women, without reference to elevated CA125 levels (2, 7).

Recurrence was observed in 6.8% women. The low LN recurrence rate further supports the notion that omitting lymphadenectomy may not substantially affect recurrence patterns in this patient group. The low LNM rates and good RFS support the notion that lymphadenectomy may not provide substantial clinical benefits in this context. Furthermore, considering the associated risks and complications of lymphadenectomy (6, 7), its routine use in all patients with low-risk endometrial cancer with elevated CA125 levels may not be justified. However, the small number of patients with recurrence limited the strength of this conclusion.

Lymphadenectomy was performed in a substantial proportion of women, reflecting the current practice of comprehensive surgical staging in many institutions, which is consistent with previous research findings (28, 29). The mean number of LNs removed was 20, suggesting a thorough evaluation of the LNs. However, despite the comprehensive LN assessment, only 3.2% exhibited LNM on pathology, as aforementioned.

Adjuvant treatment decisions within the cohort exhibited significant variability. This variability highlights the lack of consensus regarding the necessity of adjuvant therapy in this specific patient group.

Although five women (6.8%) in our cohort had endometrioid-type carcinoma grade 3 on final histology with a high CA125 level, no LNM or LN recurrence was recorded. However, one case of stump recurrence was recorded after 1 year.

To our best knowledge, no other multicenter study has examined this specific group of low-risk premenopausal women with elevated CA125 levels. Nonetheless, it is essential to acknowledge certain limitations of our study, including its retrospective nature, small sample size, and the absence of a control group. In addition, the patient group may not have been homogeneous as the data were collected over an extended period, during which medical and surgical recommendations may have varied. Obtaining long-term follow-up data would offer a more comprehensive understanding of the outcomes in this patient population. Another limitation of this study is the absence of molecular characterization, which is recommended in the most recent guidelines. This omission may impact the understanding of lymph node metastasis, particularly in relation to elevated CA 125 levels (30, 31). Therefore, we recommend future prospective studies with larger cohorts and with molecular characterization to validate our findings and provide valuable insights for clinical practice. In conclusion, in premenopausal women diagnosed with low-risk endometrial cancer and elevated CA125 levels, the likelihood of LN metastasis or recurrence is minimal and the overall prognosis is favorable. Therefore, our study’s findings suggest that routine lymphadenectomy may not be imperative in this specific group of patients and thus can be omitted. Furthermore, sentinel lymph node mapping could be considered an alternative method for assessing lymph node involvement in low-risk endometrial cancer patients as well as molecular characterizations for recurrence (30–32).

## Data Availability

The raw data supporting the conclusions of this article will be made available by the authors, without undue reservation.
